# Hemothorax from a Thoracic Chalk-Stick Fracture in Ankylosing Spondylitis: A Case Report

**DOI:** 10.5811/cpcem.47257

**Published:** 2026-03-19

**Authors:** Armin Dehkordi, Daniel Aloise, Eric Scheppke, Mary Christodoulou, Grayson Gigliotti, Tony Zitek

**Affiliations:** * Arkansas College of Osteopathic Medicine at Arkansas Colleges of Health Education, Fort Smith, Arkansas; † Mount Sinai Medical Center Miami Beach, Department of Emergency Medicine, Miami Beach, Florida; ‡ Florida International University, Herbert Wertheim College of Medicine, Miami, Florida

**Keywords:** chalk-stick fracture, ankylosing spondylitis, hemothorax, case report

## Abstract

**Introduction:**

Chalk-stick fractures are transverse spinal injuries seen in patients with ankylosing spondylitis due to chronic inflammation and spinal rigidity. These fractures may result from minor trauma and are associated with potentially fatal complications. While spinal fractures in ankylosing spondylitis are well recognized, thoracic chalk-stick fractures complicated by hemothorax from vascular injury remain exceedingly rare. We present a case of an elderly male with ankylosing spondylitis who sustained a thoracic chalk-stick fracture following a ground-level fall, complicated by hemothorax and hemorrhagic shock. This case highlights a rarely reported but life-threatening complication and emphasizes the importance of early imaging and high clinical suspicion in this high-risk population—even after minor trauma.

**Case Report:**

A 90-year-old male with known history of ankylosing spondylitis presented to the emergency department after a ground-level fall associated with syncope. He had thoracic back pain, dyspnea, and hypotension. Computed tomography revealed a thoracic vertebra 11 chalk-stick fracture with interspinous vascular injury and a large, right-sided hemothorax. The patient underwent emergent chest tube placement, blood transfusion, and vasopressor support, which initially stabilized his condition. However, his hospital course was complicated by multiple comorbidities, and he ultimately died after prolonged critical care.

**Conclusion:**

This case illustrates the potential for catastrophic vascular complications from minor trauma in patients with ankylosing spondylitis. Thoracic chalk-stick fractures may result in life-threatening hemothorax and hemorrhagic shock. Emergency physicians should maintain a high index of suspicion and obtain early radiographic imaging to facilitate timely diagnosis and intervention in this high-risk population.

## INTRODUCTION

Chalk-stick fractures are complete, transverse disruptions of the vertebral body, intervertebral disc, and posterior elements, resembling a snapped piece of chalk.^1^ They are commonly seen in patients with ankylosing spondylitis (AS), a chronic inflammatory spondyloarthropathy strongly associated with the HLA-B27 allele, where progressive enthesitis and ligamentous ossification result in a rigid yet brittle spine prone to fracture even after minor trauma.^3^,^5^–^7^ While these injuries are most common in the cervical spine, 10–11% occur in the thoracic region.^2^–^5^ Thoracic fractures in AS are particularly concerning due to their proximity to vital vascular structures. Disruption of posterior intercostal arteries, coursing on the anterolateral vertebral body, can result in hemothorax or retroperitoneal bleeding, potentially leading to hemorrhagic shock and death if not promptly recognized.^6^–^8^

We present a case of an elderly male with AS who developed a large, right-sided hemothorax after a ground-level fall caused a thoracic vertebra 11 (T11) chalk-stick fracture and interspinous vascular injury. The case highlights how these fractures can present subtly and progress rapidly. Uniquely, this injury involved the thoracolumbar junction, was initially clinically subtle, and led to catastrophic vascular injury—an uncommon but life-threatening complication in AS. This case underscores the need for a high index of suspicion, early cross-sectional imaging, and prompt coordination of hemorrhage control and definitive stabilization, even in the context of low-energy trauma.

## CASE REPORT

A 90-year-old male presented to our emergency department (ED) after experiencing a syncopal episode, resulting in a ground-level backward fall. The patient reported shortness of breath and pain in his upper back and the back of his head following the fall with brief witnessed loss of consciousness. Notably, the patient had a history of AS, hypertension, congestive heart failure, atrial fibrillation on apixaban without any rate-controlling agents, end-stage renal disease on dialysis, and polycystic kidney disease.

*CPC-EM Capsule*

What do we already know about this clinical entity?

*Chalk-stick fractures are unstable transverse spinal injuries seen in ankylosing spondylitis, often resulting from minimal trauma and associated with high morbidity and mortality*.

What makes this presentation of disease reportable?

*We describe a thoracic chalk-stick fracture with interspinous vascular injury causing massive hemothorax and hemorrhagic shock after low-energy trauma*.

What is the major learning point?

*Timely imaging and high suspicion are critical. Even minor trauma in ankylosed spines can cause catastrophic vascular injury needing rapid management*.

How might this improve emergency medicine practice?

*Being vigilant for vascular injury in ankylosing spondylitis trauma will lead to rapid imaging and emergent intervention to prevent complications*.

Upon arrival, the patient presented with the following: Glasgow Coma Scale of 15; body mass index of 29.9 kilograms per square meter; temperature of 36.8 degrees Celsius; heart rate of 71 beats per minute; respiratory rate of 20 respirations per minute; blood pressure of 88/59 millimeters of mercury (mm Hg), and oxygen saturation on pulse oximetry of 97% on room air. He appeared tachypneic with accessory muscle use. A cervical collar was placed immediately on arrival to the ED. Pertinent physical examination findings included decreased breath sounds on the right and midline thoracic spinal tenderness. The remainder of the physical examination was unremarkable. The patient became hypotensive to 75/46 mm Hg and was subsequently started on norepinephrine with improvement of blood pressure (Table 1).

Point-of-care ultrasound (POCUS) was performed to evaluate for abdominal aortic aneurysm and to assess the lungs. The initial limited exam focused on the apices and demonstrated lung sliding bilaterally, suggesting absence of pneumothorax in those regions. The inferior, lateral, and posterior lung zones were not examined at that time and, therefore, the large hemothorax was not identified on POCUS. A chest radiograph obtained shortly thereafter revealed a large, right-sided pleural effusion without visible pneumothorax, consistent with the subsequent computed tomography (CT), which demonstrated a large hemothorax and only a small anterior pneumothorax.

**Image 1:**
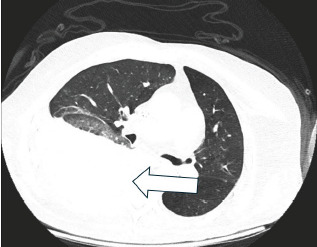
Axial computed tomography angiogram of the chest showing a large, right-sided hemothorax (arrow). Findings were secondary to an acute thoracic vertebra 11 chalk-stick fracture in the setting of ankylosing spondylitis. The fracture disrupted adjacent interspinous vasculature, resulting in hemorrhagic shock, although no active contrast extravasation was identified.

A CT angiogram of the chest, abdomen, and pelvis identified a large, right-sided hemothorax secondary to an acute chalk-stick fracture of the T11 vertebra in the setting of AS with associated interspinous vascular injury (Image 1).

**Image 2:**
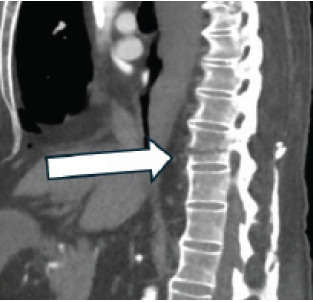
Sagittal computed tomography angiogram of the chest, abdomen, and pelvis (cropped to thoracolumbar region) highlighting an acute chalk-stick fracture (arrow) at thoracic vertebra 11 in a patient with ankylosing spondylitis. This unstable fracture at the thoracolumbar junction was associated with interspinous vascular injury and a large, right-sided hemothorax, underscoring the risk of catastrophic vascular complications in this population.

Thoracic and cardiovascular surgery were consulted and expressed concern about active extravasation of a spinal artery and an unstable thoracic spine given the chalk-stick fracture (Image 2).

A right femoral central venous catheter was placed for transfusion of two units of packed red blood cells, a right femoral arterial line was inserted, and a right-sided 36 French chest tube was placed with approximately 500 milliliters of blood return. Following chest tube placement, the patient’s work of breathing and hemodynamics improved, with stabilization of blood pressure and reduced vasopressor requirement. The patient had been on chronic anticoagulation with apixaban, but his last dose was more than 24 hours prior to presentation; therefore, pharmacologic reversal was not administered in the ED. In similar cases of life-threatening bleeding from factor Xa inhibitors, reversal agents such as andexanet alfa or four-factor prothrombin complex concentrate may be considered when immediately available and indicated. Key abnormalities included severe anemia (hemoglobin 8.1 g/L) and a markedly elevated lactate (8.0 mmol/L), consistent with lactic acidosis from profound tissue hypoperfusion in hemorrhagic shock and likely compounded by severe anemia and impaired lactate clearance from underlying end-stage renal disease. The complete laboratory panel is shown in Table 2.

The patient was admitted to the surgical intensive care unit (SICU) with a diagnosis of a chalk-stick fracture of the T11 vertebra, secondary to traumatic fall and an interspinous vascular injury resulting in a right unilateral hemothorax. Neurosurgery, nephrology, thoracic and cardiovascular surgery were involved in the patient’s multidisciplinary care throughout his prolonged SICU stay. Hospital course was complicated by subsequent right lower extremity deep venous thrombosis diagnosed by ultrasound, for which an inferior vena cava filter was placed. The patient underwent posterior thoracic vertebra 9 (T9) to first lumbar spinal instrumentation as well as video-assisted thoracoscopic surgery throughout his month-long stay in the SICU. On hospital day 31, the patient acutely decompensated. Resuscitation was initiated, resulting in return of spontaneous circulation. However, in accordance with the family’s wishes, the patient was later extubated and pronounced deceased.

## DISCUSSION

Vertebral fractures are common in elderly patients after falls, but those with AS require particular attention. Individuals with AS experience spinal fractures at rates up to four times that of the general population, with a lifelong incidence ranging from 5–15%.^4^,^9^ These fractures often occur from low-energy trauma due to a rigid, osteoporotic spine commonly referred to radiographically as a “bamboo spine.” Most affect the cervical spine, especially cervical vertebrae 5 and 6 (81.2%), while thoracic and lumbar fractures account for 10.7% and 7.8%, respectively.^5^ Despite their lower incidence, thoracic fractures carry high morbidity and mortality due to spinal instability and neurologic compromise, present in up to 67.2% of cases.^4^

Thoracic fractures, although less frequently seen in AS, carry unique risks due to the region’s proximity to critical structures, including the pleural cavity and thoracic vessels. One rare but potentially fatal complication is hemothorax, which may result from disruption of intercostal vessels or adjacent vascular structures following spinal fracture.^6^,^10^ Right-sided hemothorax is more frequently reported, likely due to the lack of protective mediastinal organs on the right, compared to the left.^8^ Although hemothorax is typically linked with blunt or penetrating chest trauma, it can be an unexpected sign of spinal injury in patients with a rigid spine, even after minor trauma.

A few case reports discuss this rare complication. One case involved a 74-year-old woman with ankylosing spinal hyperostosis who developed massive hemothorax after a fall, caused by a T11 extension-distraction fracture.^11^ Another report described a 68-year-old man with AS and a T9 fracture extending to the ribs, resulting in right-sided hemothorax and paraplegia.^12^ These cases reinforce the need for early imaging and heightened suspicion for thoracic vascular injury in AS patients. A systematic review reported a 33.3% mortality rate among patients with spinal fracture-induced hemothorax, highlighting the importance of rapid diagnosis and aggressive management.^8^

While surgical intervention was the primary approach in previous cases, there is growing evidence that selective arterial embolization can be a life-saving adjunct in cases of active bleeding. In one reported case, a 68-year-old man with AS and thoracic spine dislocation at T11 underwent urgent angiography, revealing extravasation from the intercostal artery. Successful embolization stabilized his hemodynamics, permitting delayed spinal fixation.^13^ The case highlights the potential benefit of angiography and embolization in managing hemodynamically unstable patients with spinal fractures and suspected vascular injury.

Beyond hemorrhage, chalk-stick fractures can also lead to delayed spinal epidural hematomas, reported in 0.5–1.7% of patients.^1^ Hematomas may develop hours after injury, requiring ongoing monitoring and possible repeat imaging. In unstable patients, angiography should be strongly considered to assess for active extravasation and guide timely intervention. The absence of rate-controlling medications makes the patient’s relatively normal heart rate notable in the context of hemorrhagic shock, underscoring that elderly patients may manifest shock with blunted tachycardic responses even without rate-controlling medications.^14^

Diagnosis of chalk-stick fractures can be delayed due to overlapping injuries and masked symptoms. In cases of thoracic vertebral fractures with hemothorax, respiratory compromise may overshadow signs of spinal instability, leading to a delay in definitive imaging. Standard radiographs are often insufficient due to extensive spinal fusion and overlying ossification, necessitating CT or magnetic resonance imaging for proper evaluation.^3^ Given the risks of delayed neurologic or vascular complications, a low threshold for advanced imaging is warranted in AS patients—even after minor trauma and in the absence of overt neurological deficits. Prompt recognition and intervention remain critical in optimizing outcomes and preventing devastating sequelae.

## CONCLUSION

In individuals with ankylosing spondylitis, even low-energy trauma should raise immediate concern for spinal fractures. A thorough physical examination, followed by prompt diagnostic imaging—including point-of-care ultrasound and radiographic imaging—is warranted to evaluate for structural injury. Additionally, it is essential to evaluate adjacent soft tissues, including vascular structures, even in the absence of significant pain or overt neurological deficits. A meticulous assessment of the spine, guided by a high index of suspicion, can help prevent delayed diagnosis, intervention, and associated complications. Timely recognition and appropriate management of chalk-stick fractures are critical to optimizing outcomes, particularly in patients with ankylosing spondylitis.

## Figures and Tables

**Table 1:** Key vital signs at four critical points in the emergency department course: initial presentation; transient improvement with vasopressors; subsequent decompensation; and stabilization following chest tube placement.*

Time point	HR (bpm)	RR (rpm)	BP (mm Hg)	SpO_2_ on RA	Notes
**Arrival**	71	20	88/59	97%	Tachypnea with accessory muscle use
**Initial improvement**	78	22	96/47	95%	Responded to norepinephrine
**Decompensation**	78	22	75/46	95%	Recurrent hypotension despite vasopressor support
**Post-chest tube**	92	20	124/54	96%	Improved hemodynamics and decreased need for vasopressors

* Values demonstrate relative heart rate stability despite progressive hypotension, with blood pressure improving only after definitive source control.

*BP*, blood pressure; *HR*, heart rate; *RR*, respiratory rate; *SpO_2_*, oxygen saturation; *mm Hg*, millimeters of mercury; *RA*, room air; *bpm*, beats per minute; *rpm*, respirations per minute.

**Table 2:** Lab studies of elderly patient with history of ankylosing spondylitis who presented with large, right-sided hemothorax secondary to an acute chalk-stick fracture of the thoracic vertebra 11.

Lab Test	Patient Value on Admission	Reference Range
Lactic acid	8.0 mmol/L	0.4 – 2 mmol/L
Hemoglobin	8.1 g/dL	14.0 – 18.0 g/dL
White blood cells	12.14 × 10^3^ cells/μL	4.80 – 10.80 × 10^3^ cells/μL
Red blood cells	2.75 × 10^6^ cells/μL	4.63 – 6.08 × 10^6^ cells/μL
Platelet count	295 × 10^3^ cells/μL	150 – 450 × 10^3^ cells/μL
Sodium	134 mmol/L	136 – 145 mmol/L
Chloride	93 mmol/L	98 – 107 mmol/L
BUN	28 mg/dL	7 – 18 mg/dL
Creatinine	5.9 mg/dL	0.7 – 1.3 mg/dL

*BUN*, blood urea nitrogen *dL*, deciliter; *L*, liter; *mg*, milligrams; *mmol*, millimoles; *μL*, microliter.
